# Variations in Accumulation of Lignin and Cellulose and Metabolic Changes in Seed Hull Provide Insight into Dehulling Characteristic of Tartary Buckwheat Seeds

**DOI:** 10.3390/ijms20030524

**Published:** 2019-01-26

**Authors:** Chao Song, Chengrui Ma, Dabing Xiang

**Affiliations:** 1 French Associates Institute for Agriculture and Biotechnology of Drylands (FAAB), The Jacob Blaustein Institutes for Desert Research, Ben-Gurion University of the Negev, Sede Boqer 84990, Israel; sc502632899@gmail.com; 2 College of Pharmacy and Biological Engineering, Chengdu University, Chengdu 610106, China; mcr1953@outlook.com; 3 Key Laboratory of Coarse Cereal Processing, Ministry of Agriculture and Rural Affairs, Chengdu 610106, China

**Keywords:** tartary buckwheat, seed development, dehulling efficiency, lignin, GC-MS

## Abstract

Tartary buckwheat (*Fagopyrum tataricum*) is considered a profitable crop that possesses medicinal properties, because of its flavonoid compounds. However, the dehulling issue is becoming the bottleneck for consumption of Tartary buckwheat seed. In this study, we investigated the relation between dehulling efficiency and content of lignin and cellulose in the seed hull. Moreover, the untargeted metabolomics analysis, including partial least squares discriminant analysis (PLS-DA) and principal component analysis (PCA), were performed to examine the pattern of metabolic changes in the hull of Tartary buckwheat seeds, XQ 1 and MQ 1, during seed development using gas chromatography mass spectrometry (GC-MS). In mature seed hull the accumulation of highest lignin and lowest cellulose were observed in the hull of MQ 1 seed, a dehulling-friendly variety with highest dehulling efficiency (93%), than that in other dehulling recalcitrant varieties, such as XQ 1 with a range of dehulling efficiency from 2% to 6%. During seed development, the total content of lignin and cellulose increased. MQ 1 and XQ 1 displayed a similar trend in the change of lignin and cellulose that the content was decreased in lignin and increased in cellulose. PCA result showed the metabolic differentiations between MQ 1 and XQ 1 during seed development. The results of our study suggest the compensatory regulation of lignin and cellulose deposition in the hull of mature and developing seed, and deviation of MQ 1 from the ratio of lignin to cellulose of other dehulling recalcitrant varieties may have been a contributing factor that resulted in the dehulling differentia.

## 1. Introduction

Buckwheat, which belongs to the Polygonaceae family, is a dicotyledonous crop and mainly grows in Russia and China [1]. Tartary buckwheat (*Fagopyrum tataricum*) and common buckwheat (*F. esculentum*) are the most widely grown species among the main species of the buckwheat with agricultural significance [2]. Buckwheat is considered a beneficial food component of nutritional valuable compositions and flavonoids compounds. In buckwheat, 6.82–15.02% protein was detected from the seed flour of 39 buckwheat cultivars. Besides, similar content in crude fibre on average of 2.30% was observed in the Tartary and Common buckwheat flour [3]. Interestingly, buckwheat showed the highest antioxidative activity of methanolic extracts among the cereal grain, such as barley, oat and wheat [4]. The rutin, as the main components of flavonoids in Tartary buckwheat, accounted for around 85% of total antioxidative activity [5,6]. Flavonoids were detected in all the plant organs of both Tartary and common buckwheat, including sprouts, seeds and seed hull [7,8]. In aerial parts of buckwheat plant, the highest flavonoids content was found in flowers followed by leaves and stems [9]. It has been described that the flavonoids possess medicinal properties, such as antioxidant, anti-inflammatory and anticarcinogenic [10,11]. Tartary buckwheat is attracting more research focus, because it tends to have higher contents of the specific bioactive component than common buckwheat [12,13], and shows higher antioxidant capacity than common buckwheat [14]. For example, the concentration of rutin, the predominant polyphenol in buckwheat, was 81 mg/g in the groats of Tartary buckwheat, which is much higher than in that of common buckwheat (0.2 mg) [15]. Besides, Epidemiological researches showed that Tartary buckwheat, as a significant part of the diet of people in the mountainous Liangshan region of Sichuan Province, China, reduced the occurrence of diabetes and hypertension [16].

The seed is actually a fruit, an achene of Tartary buckwheat. The hull (pericarp) tightly surrounded the testa, endosperm and embryo [17]. The dehulled Tartary buckwheat seeds are principally processed as flour for making various breads, cookies, noodles, tea and cakes [18]. However, the uses of Tartary buckwheat seeds are limited by the dehulling process. Numerous attempts have been made to improve the dehulling process. Chen et al. [19] and Liu et al. [20] optimized the huller for Tartary buckwheat seed, but only around 35% of raw seed materials were dehulled, including whole and broken groats. The dehulling is becoming the bottleneck for consumption of Tartary buckwheat seed. However, there has been no report on the cause of the dehulling issue in Tartary buckwheat seed.

Extensive researches involving the bioactive compounds in the Tartary and common buckwheat hull have been well performed regarding the improvement of extraction technique [21], the determination of antioxidative ability [22] and identification of phenolics [23]. However, only a few studies determined the chemical component in Tartary buckwheat hull. Previous research has reported that lignin and cellulose are the dominant components in common buckwheat hull, and the content of lignin and cellulose reached 31.6% and 35.6% [24]. Biel et al. evaluated chemical composition in common buckwheat seed hull and showed a similar result that seed hull consists of 34.8% and 36.5% in the content of lignin and cellulose [25]. The limited research on the hull of Tartary buckwheat seed restricts the improvement of dehulling process and the knowledge for breeding new varieties. 

Interestingly, there is a Tartary buckwheat variety, Miqiao 1, which showed a great advantage in seed dehulling [26]. Knowing the differences between Miqiao 1 and other varieties is of great potential in the breeding program to improve the dehulling ability of Tartary buckwheat seed. Therefore, the main aim of this study is to examine the main chemical component and the metabolic patterns in the hull during seed development which might be related to result in different dehulling efficiency.

## 2. Results

### 2.1. Dehulling efficiency, Lignin and Cellulose in Tartary Buckwheat Seeds

#### 2.1.1. Dehulling Efficiency in Tartary Buckwheat Seeds

**Figure 1:**
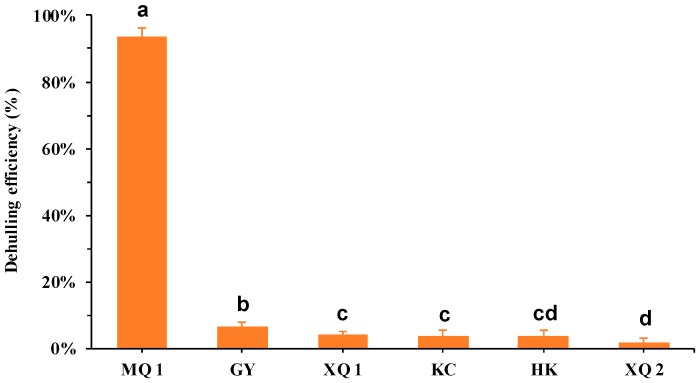
Dehulling efficiency of different Tartary buckwheat seeds. MQ 1, Miqiao 1; GY, Guyuan; XQ 1, Xiqiao 1; KC, Kuci; HK, Heiku; XQ 2, Xiqiao 2. Data are expressed as mean value ± standard deviation and sorted by dehulling efficiency value (*n* = 6). Different letters indicate significant difference between varieties (*p* < 0.05).

With the aim of determining the dehulling efficiency of Tartary buckwheat seeds, different varieties were tested in this study. In a previous study, Liu et al. [26] mentioned that Miqiao 1 (MQ 1) is the variety that can produce the unlimited edible Tartary Buckwheat rice by dehulling. In our result, MQ 1 showed the notable highest dehulling efficiency (93%) in comparison with other varieties (Figure 1). There are significant differences between the other varieties with the dehulling efficiency range from 2% (XQ 2) to 6% (GY, Guyuan), but which are still much lower than MQ 1.

#### 2.1.2. The Lignin and Cellulose Content in Seeds Hull

**Figure 2:**
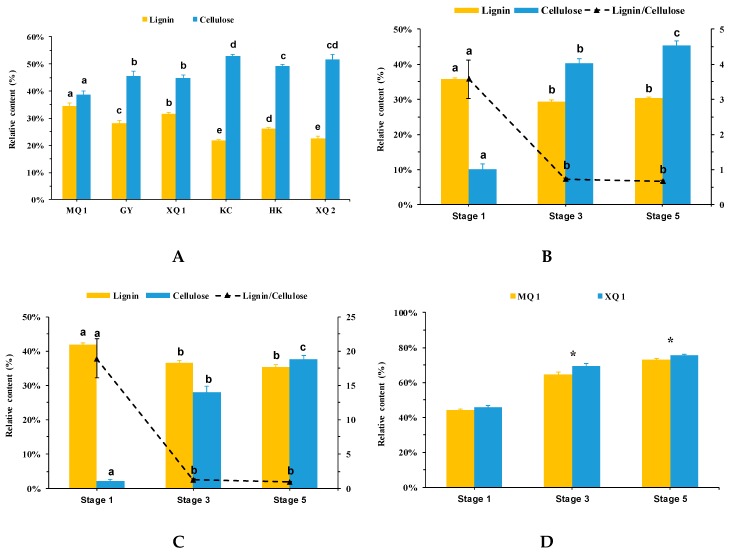
Lignin and cellulose content in the hull of Tartary buckwheat seed. S1, S3 and S5 indicate different developmental stages, stage 1, 3 and 5, of MQ 1 (M) and XQ 1 (X), respectively. (**A**) Lignin and cellulose content in the hull of mature seed (*n* = 3); (**B,C**) Lignin and cellulose content in the hull of different developmental stages of XQ 1 (**B**) and MQ 1 (**C**). Bars with different letter indicates significant differences (*p* < 0.05). (**D**) The total content of lignin and cellulose in the hull of XQ 1 and MQ 1 at each developmental stage; asterisk indicates the significant difference (*p* < 0.05) between XQ 1 and MQ 1.

As the main component of seeds hull, lignin and cellulose content of mature seeds were evaluated (Figure 2A). we observed dramatic changes in the content of lignin and cellulose across varieties. Decreases in lignin and increases in cellulose were found along with the reduction of dehulling efficiency. MQ 1, with highest dehulling efficiency, showed the highest content in lignin (35%) and the lowest content in cellulose (39%) compared with other varieties. Interestingly, no obvious differences were found in the total amount of lignin and cellulose among these varieties, with a range from 73% to 76% of the seed hull total biomass.

To gain insights into the accumulation of lignin and cellulose in the hull of developing seed, the seed hull of MQ 1 and XQ 1 at three different stages (Stage 1, 3 and 5) [27] were examined. MQ 1 and XQ 1 showed a similar trend in the change of lignin and cellulose in the hull during seed development. In the hull at stage 1, both MQ 1 and XQ 1 were observed with the accumulation of highest lignin and lowest cellulose (Figure 2B,C). Meanwhile, the ratio of lignin to cellulose showed the highest value in XQ 1 (3.6-fold) and MQ 1 (18.9-fold) at stage 1. The ratio was reduced to the lowest level in mature seed hull (Stage 5) in XQ 1 (0.67-fold) and MQ 1 (0.9-fold) during seed development. A notable switch in the ratio of lignin to cellulose occurred in the hull of XQ 1 at stage 3, showing higher cellulose than lignin, due to the increasing accumulation in cellulose content, which happened in the hull of MQ 1 at stage 5. During seed development, the total content of lignin and cellulose in the hull was increased in both MQ 1 and XQ 1 (Figure 2D). The significant differences in the total content of lignin and cellulose were observed between MQ 1 and XQ 1 of developmental stage 3 and 5.

### 2.2. Metabolic Changes in the Hull of Developing Seeds

#### Identification of Differentially Expressed Metabolites

**Figure 3:**
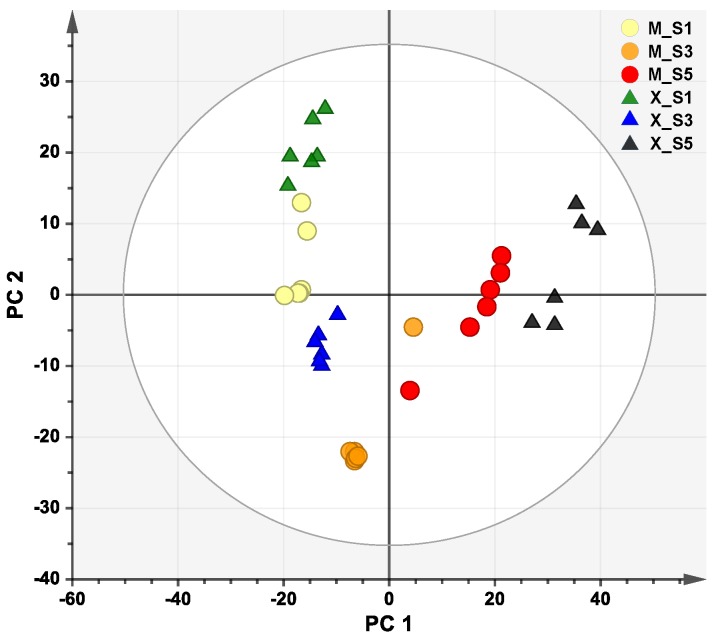
Principal component analysis (PCA) score plot. Each data point represents an independent sample. S1, S3 and S5 indicate different developmental stages, stage 1, 3 and 5, of MQ 1 (M) and XQ 1 (X), respectively. Ellipse was drew based on Hotelling’s T2 (95%).

Nine hundred and ninity-one substance peaks were extracted across 36 hull samples from XQ 1 and MQ 1 at different developmental stages. The peak area, normalized to total ion content of each sample, was imported to SIMCA-P 13.0 (Umetrics, Umeå, Sweden) to perform multivariate statistical analysis. To obtain a global overview of the metabolic changes that occurred in the hull during seed development, principal component analysis (PCA) was used to analyze and visualize the data set. PCA score plot (Figure 3) showed a clear grouping between sample groups and that first two components (PC1 and PC2) accounted for 37.9% and 18.5% of the total variables. The R2X (goodness-of-fit) and Q2 (goodness-of-prediction) of PCA are 0.900 and 0.760, which indicates the stable and reliable in the model of PCA.

To identify the differentially expressed metabolites, partial least squares discriminant analysis (PLS-DA) model, a supervised multivariable statistical analysis, was performed to identify these metabolites in the seed hull with variable of importance in projection (VIP > 1) and *t*-test (*p* < 0.05) between different developmental stages and varieties (Figure S1). The mode fitting parameters of all the comparisons were more than adequate, being goodness-of-fit R2Y ≥ 0.987 and goodness-of-prediction Q2Y ≥ 0.940 in each comparison. The significantly expressed metabolites of each comparison were collected together to create the table list, including differential expressed metabolites. In summary, a total of 22 hull metabolites were obtained, which were significantly changed during seed development in at least one of the developmental stages or between varieties (Table S1).

**Figure 4:**
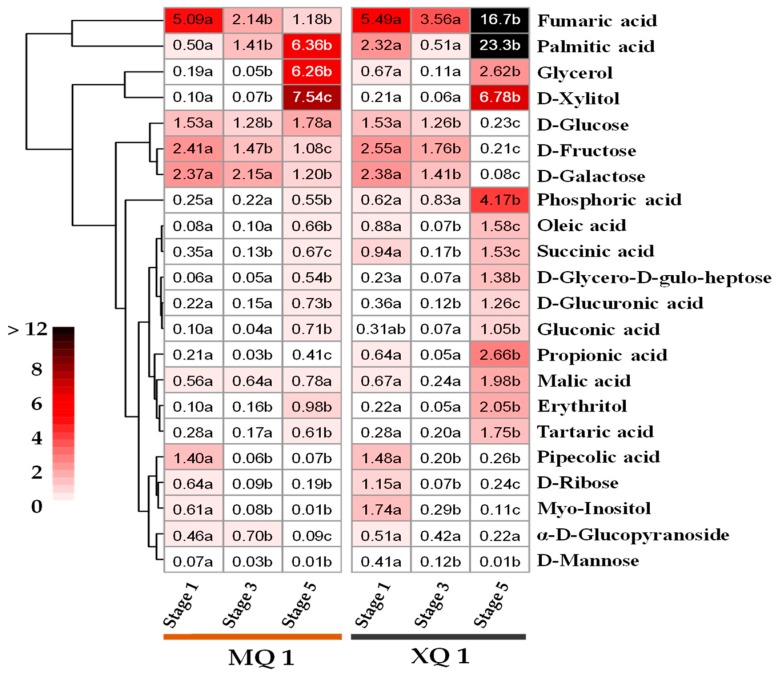
Hierarchically clustered heat map of differential expressed metabolites in the hull of different developmental stages. The relative content was normalized to the QC (quality control). The values with different letters show significant difference across the seed developmental stages both of XQ 1 and MQ 1.

The data were visualized by using a relative content, normalized to quality control (Figure 4). Heat map showed that metabolites followed a similar pattern in the change of relative content in the hull of MQ 1 and XQ 1 across seed development. For instance, myo-inositol, d-ribose and pipecolic acid were significantly decreased from developmental stage 1 to stage 3 in the hull of both MQ 1 and XQ 1. Besides, d-Glucose, d-Fructose and d-Galactose showed a decreasing trend in the hull across seed development of MQ 1 and XQ 1. However, a noticeable increase was observed in some metabolites from developmental stage 3 to stage 5, such as propionic acid, succinic acid and oleic acid. Notably, at stage 5 d-xylitol, glycerol and palmitic acid were highly accumulated in the hull of MQ 1 and XQ 1. In the hull of XQ 1 at stage 5, fumaric acid, an intermediate in the citric acid cycle, and palmitic acid, a saturated fatty acid, were increased with relative content of 16.7 and 23.3. Compared with MQ 1 at stage 5, the significant lower content in sugar, including d-Glucose, d-Fructose and d-Galactose in the hull of XQ 1 were found with 0.13-fold, 0.19-fold and 0.07-fold, respectively. On the other side, in the hull of XQ 1 at stage 5 some metabolites, such as fumaric acid, myo-inositol and phosphoric acid, showed much higher content than those in the hull of MQ 1, being 14.1-fold, 7.2-fold and 7.5-fold (Table S2).

## 3. Discussion and Conclusion

To our knowledge, this is the first study to explore the association between the dehulling efficiency and the change of main chemical component in the hull of the mature and developing seed of Tartary buckwheat. In order to obtain insight into the metabolic changes in the hull during seed maturation, we performed metabolic analysis for identifying differentially expressed metabolites. In this study, Miqiao 1 (MQ 1) showed a notable dehulling efficiency among the varieties. On the other side, our results showed lower dehulling efficiency in other varieties than reported in previous research [19,20]. By boiling and steam cooking seed at high temperature or soaking seeds in the warm water, the starch tissue was hardened, and the hull was softened, due to the moisture in the hull [28]. To a certain degree, the current dehulling method run on the industrial level that combines preprocesses with grinding, can increase the dehulling efficiency. However, the steaming grains of Tartary buckwheat by the current dehulling method is linked with enormous nutrition losses and energy expense [29]. 

Lignin and cellulose are the most abundant organic carbon on the earth. Lignin plays a vital role in the structural integrity of the cell wall and strength and stiffness of plant stem [30,31]. Cellulose microfibrils give tensile strength to cell walls. Moreover, lignin encasing the cellulose microfibrils imparts rigidity to cell walls [32]. The positive correlation was found between the accumulation of lignin and breaking strength [33,34]. Genet et al. [35] reported that the increase in cellulose content could be related to the increased tensile strength in the root. Taken together, these studies may indicate that, in the tissue of great content in lignin and cellulose, lignin competes with cellulose for showing the main characteristic of hardness or tenacity in tissue. In this study, the significant lower lignin and higher cellulose in content were found in the seed hull of Tartary buckwheat varieties (dehulling recalcitrant) which showed much lower dehulling efficiency in comparison with MQ 1 (dehulling friendly) (Figure 1 and Figure 2A). MQ 1 somewhat deviated from the ratio of lignin to cellulose of other dehulling recalcitrant varieties. It may have been a contributing factor that resulted in the dehulling differentia considering the function of lignin and cellulose in cell mentioned above. In previous studies, Lignin and cellulose were determined as the main chemical components in the hull of common buckwheat [24,25]. In our case, we observed that the seed hull of Tartary buckwheat is mainly composed of lignin and cellulose, concomitant with a total amount from 73% to 76% in the hull of mature seed. The total contents of lignin and cellulose were unaffected by the different varieties, but the ratio of lignin to cellulose in the hull showed significant difference among varieties (Figure S2). This phenomenon could be attributed to the compensatory regulation of lignin and cellulose deposition. This regulation was observed in transgenic aspen trees that the reduced lignin was compensated by a concomitant increase in cellulose [36]. The similar compensatory regulation was also found during seed development of MQ 1 and XQ 1. During seed development, the total content of lignin and cellulose was increased in the hull of MQ 1 and XQ 1, which was consistent with the seed elongation published in our previous work [27]. Comparing with MQ 1, the lower accumulation of lignin was compensated by accumulating higher content in cellulose in XQ 1 (Figure 2B,C). This compensatory regulation of lignin and cellulose is associated with the changes in radial auxin gradient for tension wood formation in woody plants [37,38,39]. Taken together, this suggested the interaction between auxin, lignin and cellulose in the hull during seed development, and provides a direction for future research in the dehulling issue of Tartary buckwheat seed.

According to the PCA score plot based on the extraction of substance peaks, each sample group was separated. The separation of sample groups indicated that seed development led to great differentiation in metabolism. Moreover, the samples from stage 1 and stage 3 were also clearly separated between MQ 1 and XQ 1. It showed the metabolic variations in the seed hull during seed development of MQ 1 and XQ 1 and suggested that early stages of seed development differentiated MQ 1 from XQ 1 in dehulling efficiency. The switch in the ratio of lignin to cellulose, from stage 1 to stage 3, could also be evidence of differentiation of metabolic changes between MQ 1 and XQ 1. In all, the metabolic analysis provided a relevant basis for clarifying various changes of the chemical component in the seed hull during seed development. But more explorations, such as phytohormone, tissue anatomy, could be taken into consideration in future research to reveal the causation of dehulling issue during seed development of Tartary buckwheat seed. For instance, in soybean the structural differentia, including thickened seed coat tissues [40], cuticular cracks [41] and a prominent light line of the palisade cells [42], was proposed to account for the hard and soft seeds regarding the different permeability of the seed coats to water [43]. The structural character of hull or seed coat is related to its function. Thus, the anatomical research on the hull of Tartary buckwheat seeds could be a field of great potential to account for the dehulling issue. Knowing the detailed chemical and structural description of Tartary buckwheat seed hull during seed development will provide the basis for further dehulling studies and might assist in enriching germplasm with dehulling-friendly varieties.

## 4. Materials and Methods

### 4.1. Plant Materials

Six different varieties of Tartary buckwheat (F. *tataricum*) were used in this study, and the seeds were provided by the National Research and Development Center for Coarse Cereal Processing, Chengdu, Sichuan Province, China. The experiment was conducted in September-December 2014 and 2015 at Chengdu University (30°65′N and 104°19′E), Sichuan Province, China. The seeds were sown in September and harvested in December 2014 and 2015. Seedlings were thinned to final density (7 × 10^5^ ha^−1^ plants) 15 d after germination. The synthetic fertilizer (N:P:K = 15:15:15) was applied as basal fertilizer at the rate of 300 kg ha^−1^. In 2014, the hull of mature seeds of six varieties was collected using dehuller for dehulling efficiency test, cellulose and lignin content analysis. XQ 1 and MQ 1 of six varieties were selected and grown in 2015. Seeds of XQ 1 and MQ 1 at three different developmental stages (Stage 1—seed formation start; stage 3—milk-ripe stage; stage 5—mature seed) in 2015 were collected and immediately put into liquid nitrogen. Before analysis, frozen samples stored at −80 °C were freeze-dried to separate the hull for extraction. 

### 4.2. The Dehulling Efficiency

The dehulling was done by using a laboratory dehuller (DM-WZ125-II, Changhong, Co. Ltd., Cangzhou, China). The total seed number and the seed number without hull were counted from 50 g of 2 kg processed seeds. The dehulling efficiency is defined as the ratio of number of seeds without any hull residues to the total number of seeds. Seeds counting were repeated six times for estimating the dehulling efficiency.

### 4.3. Analysis of Cellulose and Lignin

The ground hull tissue of six varieties and different developmental stages of XQ 1 and MQ 1 were used for the measurement of cellulose and Lignin content. Lignin content determination was conducted based on the description in Park et al. [44]. Briefly, 0.3 g of ground samples (W_0_) were transferred into screw-top high-pressure tubes. 4 mL of 72% H_2_SO_4_ was added to the tube followed by incubation at 30 °C and 100 rpm for 60 min. After incubation, 111 mL of deionized water was added to the tube to dilute the acid concentration to 4% before hydrolysis in the autoclave at 121 °C for 60 min. Then, vacuum filter was applied to collect the lignin residue using pre-ash filtering crucible (G_0_). The weight (G_1_) of the crucible containing lignin residue was recorded after kept at 105 °C for 4 h. The furnace was used to burn the crucible with residue for calculating the ash weight (G_2_). The acid detergent lignin (%) was calculated as:

Lignin (%) = (G_1_ − G_0_ − G_2_)/W_0_ × 100%. (1)

The cellulose content was determined according to Van et al. [45]. 0.3 g of ground sample (W_1_) was transferred into a conical breaker. 0.5 g of Na_2_SO_3_ and 100 mL of acid solution, containing 2% of CTAB (Hexadecyl trimethyl ammonium Bromide) dissolved in 4% of H_2_SO_4_, were added to the conical breaker. The mixture solution was maintained at the reflux temperature for 1 h. Pre-ash filtering crucible (G_2_) combined with vacuum filter was used to collect residues rinsed with boiling water and some acetone. The weight (G_3_) of the crucible with residue was recorded after kept at 105 °C for 4 h. The furnace was used to burn the crucible with residue for calculating the ash weight (G_4_). The acid detergent cellulose (%) was calculated as:

Cellulose (%) = (G_3_ − G_4_ − G_2_)/ W_1_ × 100% − Lignin (%). (2)

### 4.4. Metabolic Profiling

For GC-MS analysis, the seed hull of XQ 1 and MQ 1 at different developmental stages were used for extraction. 50 mg of ground tissue was transferred to a 1.5 mL Eppendorf tube. 800 μL of 100% methanol was added to the tube followed by sonicating for 90 s, vortex for 30 s and centrifuging at 12,000 rpm for 15 min. 200 μL of supernatant from each sample was collected into a glass derivative bottle and dried in a vacuum concentrator (Eppendorf Concentrator Plus). 30 μL of methoxylamin priding (20 mg/L) was added to each derivative bottle and shaken for 30 s for processing oximation reaction at 37 °C for 90 min. Finally, 30 μL of derivatization reagent, N, O-bistrifluoroacetamide (containing 1% trimethylchlorosilane), was added and left reacting for 1 h at 70 °C. 

The GC-MS analysis was conducted using a 7890A/5975C GC-MS system (Agilent Technologies, Santa Clara, CA, USA) at Shanghai Sensichip Infotech Co. Ltd. (Shanghai, China). The GC-MS condition, data pre-process and metabolites detection were performed as described in published research [46]. Briefly, Principal component analysis (PCA) and partial least squares discriminant analysis (PLS-DA) model were performed to analyze normalized data set and search differential expressed metabolites with VIP (variable importance in the projection) values > 1, combined with *t*-test (*p* < 0.05) in the NIST databases (http://www.nist.gov/index.html).

### 4.5. Data Analysis 

One-way ANOVA was performed on the difference of cellulose and lignin content in the seed hull between different varieties and developmental stages using JMP 13 (SAS Institute, North Carolina, USA). Statistically significant difference was identified by a Tukey-Kramer and Student’s *t*-test at *p* < 0.05. The relative content of differential metabolites, normalized to QC (quality control), were used to build heatmap using R software (https://www.R-project.org/).

## Supplementary Materials

Supplementary materials can be found at http://www.mdpi.com/1422-0067/20/3/524/s1.
