# Supplementary material for: Variations in Accumulation of Lignin and Cellulose and Metabolic Changes in Seed Hull Provide Insight into Dehulling Characteristic of Tartary Buckwheat Seeds
Source: Int J Mol Sci. 2019 Jan 26;20(3):524. doi: 10.3390/ijms20030524 (PMC6387337; doi:10.3390/ijms20030524)
Supplement: Supplementary file 1 [file ijms-20-00524-s001.pdf]

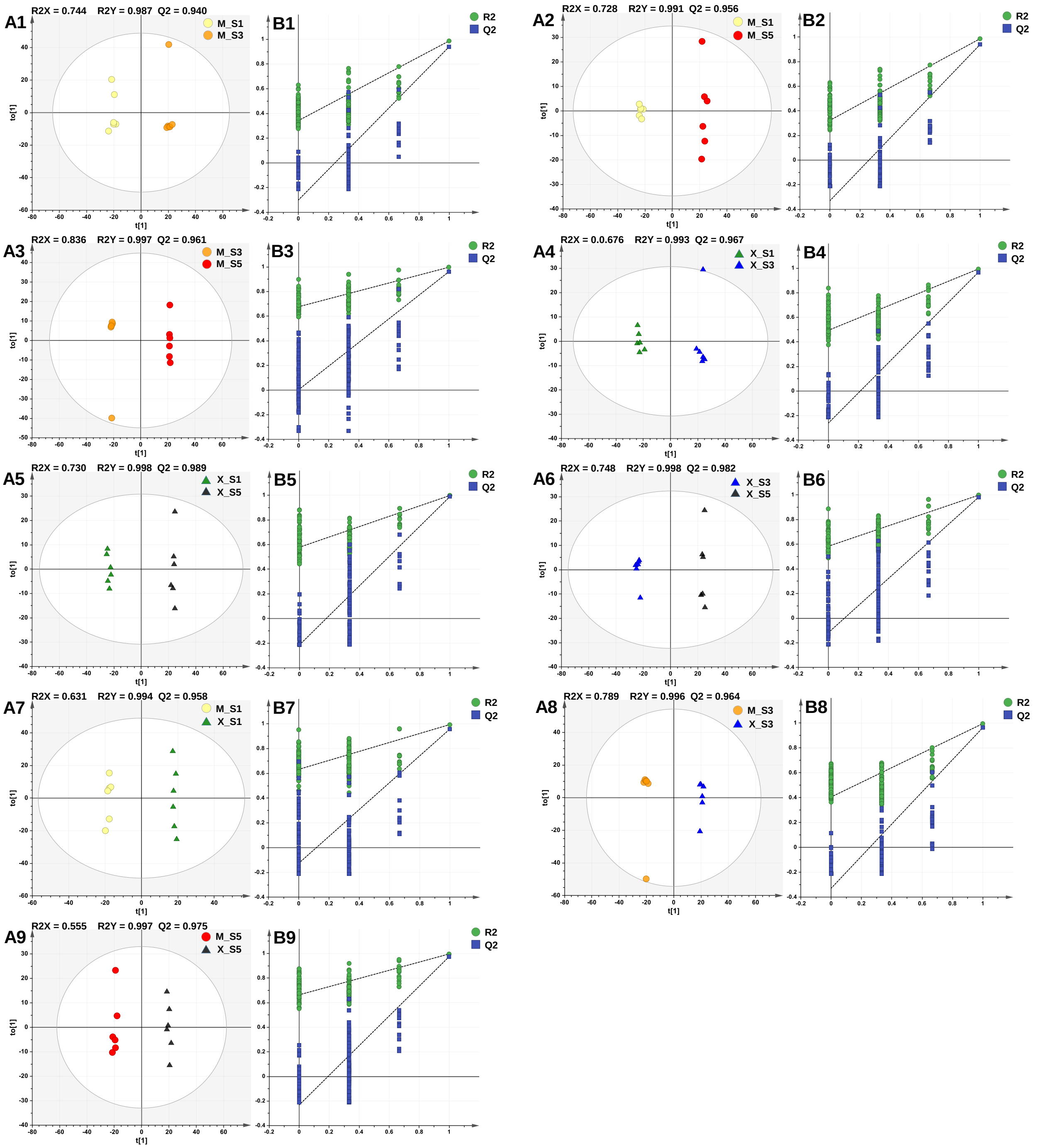

Figure S1. PLS-DA score plots and permutation test for the model discriminating hull samples from different developmental stages of Tartary buckwheat seeds. S1, S3 and S5 indicate developmental stage 1, 3 and 5 of MQ 1 (M) and XQ 1 (X), respectively. (A1-A9) PLS-DA score plots. Each data point represents an independent sample; Ellipse was drew based on Hotelling's T2 (95%); (B1-B9) a 200-times permutation test for the corresponding model.

Table S1. The differentially expressed metabolites in the hull at least one of the developmental stages of XQ 1and MQ1 seeds.

NO	Metabolite	m/z	MQ 1			XQ 1			S1	S3	S5
			S1-S3 [a]	S1-S5	S3-S5	S1-S3	S1-S5	S3-S5	XQ 1- MQ 1		
1	Oleic acid	54	NS	1.18451^{***,b}	1.14002**	1.13442***	*	1.10386***	1.41374***	NS	1.11159**
2	α-D-Glucopyranoside	54	NS	1.23367***	1.18357***	NS	NS	**	NS	NS	*
3	Succinic acid	56	*	**	1.30276***	1.29571***	*	1.13068***	1.5169***	NS	1.18395**
4	Malic acid	58	NS	NS	NS	1.22925***	1.05565***	1.12803***	NS	1.40143***	1.19593**
5	Propionic acid	66	1.06251**	*	1.30644***	1.283***	1.05888***	1.12461***	1.46829***	NS	1.3144***
6	Fumaric acid	68	1.10517**	1.09115***	1.26932***	NS	*	*	NS	NS	1.09826**
7	Erythritol	69	NS	1.20905***	1.29482***	1.17174***	1.17967***	1.18654***	1.11147*	1.2409***	1.22867**
8	Pipecolic acid	84	***	1.07696***	NS	1.24839***	1.17586***	NS	NS	1.25207***	1.09725**
9	D-Fructose	99	1.38642***	***	*	1.02003**	***	***	NS	*	***
10	D-Ribose	100	***	1.06391**	1.04944**	1.31441***	1.23957***	1.05224***	1.3684**	NS	NS
11	Glycerol	101	**	1.18171***	1.27949***	1.30364***	1.0978***	1.14992***	1.59966***	*	1.16934**
12	D-Xylitol	103	NS	1.22291***	1.31904***	1.15446***	1.07738***	1.07721***	1.235**	NS	NS
13	Palmitic acid	117	NS	1.06815**	*	1.06497**	1.1115***	1.12588***	1.3437**	NS	1.23952**
14	D-Galactose	178	NS	1.09747***	NS	1.10191***	1.23315***	***	NS	NS	1.30374***
15	Myo-Inositol	191	***	1.29207***	NS	1.31246***	1.25469***	*	1.64243***	1.02521*	*
16	D-Glucose	208	**	NS	*	*	1.21142***	***	NS	NS	1.38849***
17	Phosphoric acid	217	NS	1.08417***	1.09595**	NS	1.23517***	1.2253***	1.20326**	1.27653***	1.49153***
18	Tartaric acid	219	NS	*	1.05622**	NS	1.16591***	1.17528***	NS	NS	1.31486***
19	D-Glucuronic acid	292	NS	1.17646***	1.31315***	*	1.17842***	1.22626***	NS	NS	1.28086***
20	Gluconic acid	292	*	1.23664***	1.34742***	*	NS	*	*	NS	NS
21	D-Glycero-D-gulo-heptose	331	NS	1.15326***	1.23892***	NS	1.15429***	1.20704***	NS	NS	1.33172***
22	D-Mannose	387	*	1.15578***	NS	*	**	1.11543***	1.16238*	1.18825**	NS

[a] S1, S3 and S5 indicate the developmental stage 1, 3 and 5 of MQ 1 (M) and XQ 1 (X), respectively; [b] Variable of importance in projection (VIP) was displayed with threshold of 1; *, ** and *** indicate the significant difference in corresponding comparison at 0.05, 0.01 and 0.001 level, respectively; NS indicates no significant difference.

Table S2. The relative content of differentially expressed metabolites in the hull of different developmental stages of XQ 1and MQ1 seeds.

NO	Metabolite	MQ 1			XQ 1			Fold change (XQ 1/ MQ 1)		
		**Stage 1**	**Stage 3**	**Stage 5**	**Stage 1**	**Stage 3**	**Stage 5**	**Stage 1**	**Stage 3**	**Stage 5**
1	Oleic acid	0.0894a	0.1098a	0.6617b	0.8821a	0.0725b	1.5823c	9.8591*	0.6608^{NS}	2.3912*
2	α-D-Glucopyranoside	0.4669a	0.7048b	0.0990c	0.5185a	0.4243a	0.2293a	1.1105^{NS}	0.6020^{NS}	2.3165*
3	Succinic acid	0.3516a	0.1375b	0.6788c	0.9484a	0.1773b	1.5336c	2.6971*	1.2893^{NS}	2.2591*
4	Malic acid	0.5640a	0.6411a	0.7809a	0.6755a	0.2440a	1.9820b	1.1977^{NS}	0.3806*	2.5379*
5	Propionic acid	0.2166a	0.0323b	0.4107c	0.6496a	0.0546a	2.6676b	2.9983*	1.6907^{NS}	6.4945*
6	Fumaric acid	5.0951a	2.1454b	1.1879b	5.4907a	3.5675a	16.773b	1.0776^{NS}	1.6628^{NS}	14.1200*
7	Erythritol	0.1077a	0.1695b	0.9815b	0.2287a	0.0518a	2.0565b	2.1225*	0.3060*	2.0951*
8	Pipecolic acid	1.4054a	0.0682b	0.0779b	1.4870a	0.2087b	0.2626b	1.0580^{NS}	3.0600*	3.3679*
9	D-Fructose	2.4125a	1.4708b	1.0828c	2.5586a	1.7690b	0.2108c	1.0605^{NS}	1.2027*	0.1947*
10	D-Ribose	0.6477a	0.0935b	0.1965b	1.1549a	0.0786b	0.2489c	1.7830*	0.8405^{NS}	1.2664^{NS}
11	Glycerol	0.1995a	0.051b	6.2614b	0.6739a	0.1121a	2.6252b	3.3773*	2.1963*	0.4192*
12	D-Xylitol	0.1092a	0.0735b	7.5435b	0.2123a	0.0649a	6.7849b	1.9430*	0.8833^{NS}	0.8994^{NS}
13	Palmitic acid	0.5006a	1.4136b	6.3609b	2.3290a	0.5101a	23.313b	4.6525*	0.3608^{NS}	3.6651*
14	D-Galactose	2.3761a	2.1512a	1.2029b	2.3800a	1.4160b	0.0876c	1.0016^{NS}	0.6582^{NS}	0.0728*
15	myo-Inositol	0.6116a	0.0852b	0.0158b	1.7462a	0.2992b	0.1148c	2.8551*	3.5106*	7.2489*
16	D-Glucose	1.5377a	1.2887b	1.7878a	1.5328a	1.2608b	0.2377c	0.9968^{NS}	0.9783^{NS}	0.1329*
17	Phosphoric acid	0.2570a	0.2248a	0.5582b	0.6226a	0.8322a	4.1745b	2.4226*	3.7008*	7.4772*
18	Tartaric acid	0.2880a	0.1715a	0.6126b	0.2875a	0.2019a	1.7574b	0.9983^{NS}	1.1768^{NS}	2.8685*
19	D-Glucuronic acid	0.2213a	0.1585a	0.7359b	0.3615a	0.1228b	1.2678c	1.6338^{NS}	0.7749^{NS}	1.7228*
20	Gluconic acid	0.1072a	0.0474a	0.7194b	0.3182ab	0.0763a	1.0576b	2.9662*	1.6079^{NS}	1.4699^{NS}
21	D-Glycero-D-gulo-Heptose	0.0609a	0.0574a	0.5475b	0.2382a	0.0701a	1.3809b	3.9109^{NS}	1.2211^{NS}	2.5221*
22	D-Mannose	0.0792a	0.0333b	0.0093b	0.4131a	0.1222b	0.0137b	5.2139*	3.6680*	1.4667^{NS}

The value carrying different letters indicate the significant difference across the seed development of XQ 1 and MQ 1; '*' indicates the significant difference between XQ 1 and MQ 1 at corresponding developmental stage at 0.05 level; NS indicates no significant difference ($p > 0.05$).

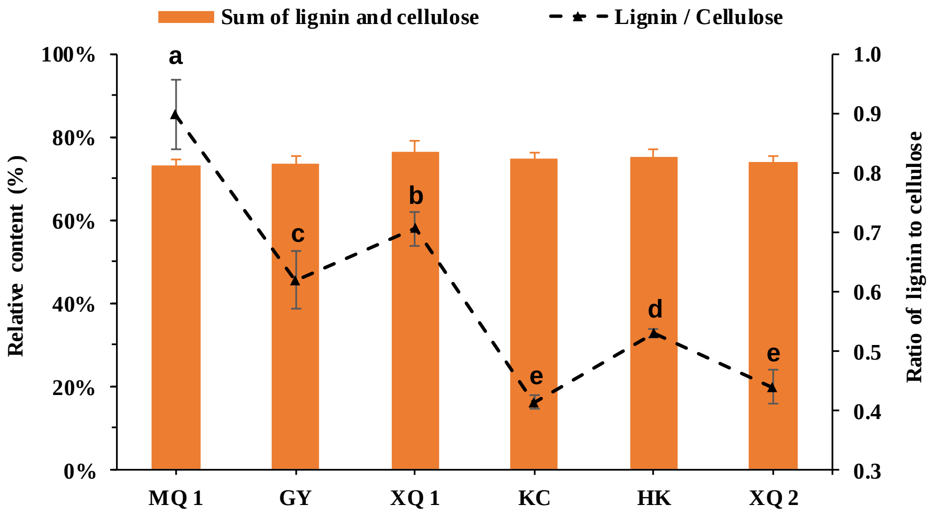

Figure S2. The sum of lignin and cellulose and ratio of lignin to cellulose in the hull of Tartary buckwheat seeds. MQ 1, Miqiao 1; GY, Guyuan; XQ 1, Xiqiao 1; KC, Kuci; HK, Heiku; XQ 2, Xiqiao 2. Data are expressed as mean value ± standard deviation and sorted by dehulling efficiency value (n = 3). Different letters indicate significant difference between varieties ($p < 0.05$).
